# Total Synthesis and Late-Stage C–H Oxidations of *ent*-Trachylobane Natural Products

**DOI:** 10.1002/anie.202113829

**Published:** 2021-11-27

**Authors:** Lukas Anton Wein, Klaus Wurst, Thomas Magauer

**Affiliations:** Institute of Organic Chemistry and Center for Molecular Biosciences, Leopold-Franzens-University Innsbruck, Innrain 80-82, 6020 Innsbruck (Austria); Institute of General, Inorganic and Theoretical Chemistry, Leopold-Franzens-University Innsbruck, Innrain 80-82, 6020 Innsbruck (Austria); Institute of Organic Chemistry and Center for Molecular Biosciences, Leopold-Franzens-University Innsbruck, Innrain 80-82, 6020 Innsbruck (Austria)

**Keywords:** Biomimetic synthesis, C–H activation, Natural products, Terpenoids, Total synthesis

## Abstract

Herein, we present our studies to construct seven ent-trachylobane diterpenoids by employing a bioinspired two-phase synthetic strategy. The first phase provided enantioselective and scalable access to five ent-trachylobanes, of which methyl ent-trachyloban-19-oate was produced on a 300 mg scale. During the second phase, chemical C–H oxidation methods were employed to enable selective conversion to two naturally occurring higher functionalized ent-trachylobanes. The formation of regioisomeric analogs, which are currently inaccessible via enzymatic methods, reveals the potential as well as limitations of established chemical C–H oxidation protocols for complex molecule synthesis.

***T*** he *ent*-trachylobane diterpenoids possess a unique [3.2.1.0^2,7^]cyclooctane subunit and a diverse oxidation pattern as exemplified by *ent*-3β-acetoxy-trachyloban-19-al (**1**), ciliaric acid (**2**), 11-oxo-*ent*-trachyloban-19-oate (**3**) and mitrephorone C (**4**) (Figure 1).^[1]^ Owing to their complex molecular framework and biological activities, the mitre-phorones have recently attracted great attention from the synthetic community.^[2–4]^ Aside from these reports, syntheses of higher oxidized *ent*-trachylobanes are still rare^[5]^ and the entire family has remained largely untouched by synthetic chemists. In line with the two-phase biosynthesis of terpenoids,^[6]^ the hydrocarbon *ent*-trachylobane (**5**) and its acid derivative **6**,^[7]^ respectively, should serve as the pivotal substrates (Cyclase Phase) for the subsequent divergent oxidation pathways (Oxidase Phase). In 2020, Renata and co-workers^[4]^ elegantly mimicked this concept by developing a semi-synthetic, chemoenzymatic approach to nine natural diterpenoids including **6** and mitrephorone A, B and C (**4**). For the enzymatic oxidation of **6**, exclusive hydroxylation at C-2 and C-7 (highlighted in red) was observed. Our continuing interest to apply the two-phase concept to several terpenoid families inspired us to study the innate reactivity of **5** and minimally oxidized derivatives thereof by means of chemical C–H oxidations methods.^[8]^ Herein, we report our findings that for the trachylobane framework non-directed, chemical C–H oxidations preferentially occur at C-11 and C-15.

To gain access to the required substrates, we modified the cyclase phase of our recently developed synthesis of mitrephorone B^[3]^ (Scheme 1). Readily available **9**, having already two of four quaternary stereocenters installed, was hydrogenated on a 6 g scale (Pd/C, H_2_) in an aprotic solvent (EtOAc) to provide decalin **10**. The use of ethyl acetate was crucial as an unexpected overreduction of the ketone occurred in alcoholic solvents such as MeOH or EtOH. Next, a two-step Robinson annulation protocol was employed to install the C-ring providing **11** ^[9]^ as a single diastereomer (C-8) in 41% yield over two steps. Methylation in the α-position (C-16) proceeded smoothly and delivered the product as single diastereomer in 81% yield. Since direct vinylation under Buchwald’s conditions (vinyl bromide, Pd_2_dba_3_, NaO*t*-Bu, DavePhos)^[10]^ resulted in complex product mixtures, a two-step procedure^[11]^ was employed.

Gratifyingly, the alkylation/aldol sequence proved to be highly diastereoselective and furnished vinylketone **12** in excellent yield. A subsequent Luche reduction of enone **12** delivered the corresponding allylic alcohol also in good yields (76%). Initial attempts to eliminate the allylic alcohol under Brønsted acidic conditions wereplagued by the formation of triene **13** together with inseparable byproducts. After extensive screening of dehydration protocols (Tf_2_O, SOCl_2_, Martin sulfurane, *p*-TsOH), elimination with Burgess reagent^[12]^ offered the only solution to provide triene **13** in 40% yield and unidentified polar byproducts. With sufficient quantities of triene **13** in hand, a thermal (175 °C) intramolecular Diels-Alder reaction delivered the desired tricyclo[3.2.1.0^2,7^]octene **14** in 85% yield together with unreacted starting material (5%). Full conversion was not observed even at higher temperatures (190 °C) and under extended reaction times (24 h). From that point on, Shenvi’s^[13]^ hydrogen atom transfer (HAT) protocol (Mn-(dpm)_3_, PhSiH_3_, *t*-BuOOH) was conducted on a 300 mg scale to deliver methyl *ent*-trachyloban-19-oate (**15**) with perfect diastereoselectivity and in excellent yield (99%). Alternatively, ester **14** was demethylated with sodium ethane thiolate to quantitatively yield the natural product *ent*-trachyloban-9-en-19-oate (**16**).^[14]^ Further diversification of methyl ester **15** was accomplished by demethylation to carboxylic acid **6** ^[15]^ or by reduction with Red-Al^®^ yielding alcohol **17**. Deoxygenation of *ent*-trachyloban-19-ol (**17**) to *ent*-trachylobane (**5**) was then realized in two steps involving Appel reaction (PPh_3_, I_2_) of the neopentylic alcohol and subsequent reduction (Pd/C, H_2_).

Having prepared ample amounts of *ent*-trachylobanes 1**5** and **6**, we turned our attention to undirected C–H-oxidation protocols (Scheme 2).^[8,16]^ To begin with, a solution of methyl ester **15** in dichloromethane was treated with methyl (trifluoromethyl)dioxirane (TFDO)^[17]^, which has frequently been used as a benchmark reagent. Interestingly, C-11 ketone **18** was observed as the main product in 43% yield without detectable amounts of regioisomeric products. Similarly, when **15** and carboxylic acid **6** were subjected to Baran’s electrochemical conditions, preferred oxidation at C-11 and not C-2 was observed. This provided direct access to the naturally occurring 11-oxo-*ent*-trachyloban-19-oate (**3**)^[18]^ and its methyl ester analog **18**. The oxidation of **15** to **18** was also accompanied by ring-opening/fragmentation to give enone **20** in 10% yield (see Supporting Information for mechanistic insights). In contrast, Baran’s ammonium ylide^[19]^ mediated electrochemical oxidation exclusively led to decomposition. When employing Ru(TMP)(CO) (**21**)^[20]^ as the catalyst for the C–H oxidation, the C-11 ketones **3** (20%) and **18** (27%) as well as C-11 alcohol **19** (38%) were isolated. The use of the more reactive Ru(TPFPP)(CO)^[21]^ led to complex product mixtures without detectable amounts of **3** or **18**, respectively. Similar results were obtained when **6** was subjected to Alexanian’s C–H chlorination protocol.^[22]^ Employing White’s iron and manganese catalyzed oxidation conditions (M^II^(PDP) or M^II^(CF_3_-PDP), AcOH, H_2_O_2_)^[23]^ led to trace amounts of a complex product mixture and decomposition of the starting material.

We assume that C-15 oxidation followed by cyclopropane opening occurs to unlock several decomposition pathways. Evidence for the C-15 oxidation was obtained by applying Costas’ modification ((*R*,*R*)-Mn(CF_3_-PDP) (**24**), TfOH, TFE).^[24]^ In this case, we observed C-15 oxidation followed by ring-opening to give the alcohol **22** ^[25]^ in 30% yield (see Supporting Information for mechanistic insights). Importantly, alcohol **22** comprises the *ent*-atisane framework (**23**) and might be used as an entry point for several members of this family.^[26]^ In contrast, the enzymatic oxidation bypasses this intrinsic ring-opening preference as various C-15 oxidized trachylobanes have been isolated from natural sources.^[27]^

Coordination of the free acid **6** to the catalyst was initially expected to direct the C–H oxidation away from C-11 to reach C-2 and C-6.^[28]^ However, the axially aligned carboxylic acid may suffer from steric hindrance thus preventing proper coordination and regiocontrol (compare with X-ray crystal structure of **6** in Scheme 1). The selectivity for the C-11 position was rationalized by the electronically activating cyclopropane unit and the deactivating carboxylic acid/ester group. To our surprise, even alcohol **17** undergoes electrochemical C–H oxidation at C-11, albeit in very low yields. When *ent*-trachylobane (**5**) was subjected to the previously investigated conditions, only decomposition was observed. The observed site-selectivities of chemical methods for C-11 and C-15 are opposite from those obtained for enzymatic methods. Due to geometrical constraints of the enzymes, exclusive oxidation of C-2 and C-7 is observed and oxidation of C6 only occurs after oxidation at C-7. While highly reactive TFDO, electrochemical oxidation and Ru(TMP)(CO) (**21**) select for the C-11 methylene, the steric topology of the bulky (*R*,*R*)-Mn(CF_3_-PDP) (**24**) matches the C-15 position and could therefore overcome that intrinsic bias (see Supporting Information for further details). Noteworthy, the use of (*S*,*S*)-**24** did not allow for any selectivity change but mostly led to decomposition.

To complete our studies, we turned our attention toward some directed oxidation methods.^[29]^ To begin with, we investigated the Suárez oxidation protocol^[30]^ employing alcohol **17**. Under these conditions, the C-20 iodide **25** was obtained in 28% yield as the sole product. To the best of our knowledge, C-20 oxidized *ent-*trachylobane natural products have not been isolated thus far, rendering **25** and potential derivatives thereof valuable targets for future structure-activity relationship (SAR) studies.^[31]^

Next, quantitative oxidation (PCC) of alcohol **17** followed by the formation of imine **26** set the stage for the Schönecker oxidation protocol.^[32]^ Exposure of **26** to copper (II) triflate in the presence of oxygen delivered the C-3 alcohol together with an inseparable mixture of byproducts. From this mixture, we were able to isolate its naturally occurring acetoxy derivative **1** ^[33]^ in 18% yield over two steps. Elimination of the alcohol or intermediates during the oxidation seemed to prevent higher yields for **1**. In an attempt to address this issue, we replaced the 2-picolylamine with the more electron-rich 4-methyl-2-picolylamine and copper(II) triflate with copper(I) hexafluorophosphate as reported by Baran.^[34]^ However, this measure was found to be detrimental and gave even lower yields for **1**. Nevertheless, subjecting imine **26** to the third generation conditions (copper(II) nitrate trihydrate and hydrogen peroxide)^[35]^ resulted in almost doubled yield over two steps (32%). Efforts to enable C-18 functionalization by employing Yu’s protocol remained unsuccessful.^[36]^

In summary, we have developed an enantioselective and scalable total synthesis of seven naturally occurring *ent*-trachylobanes. The cyclase phase enabled assembly of the *ent*-trachylobane carbon framework on a 300 mg scale. Investigation of a variety of undirected and directed aliphatic C–H oxidation methods in the oxidase phase culminated in the first total synthesis of the C-11 oxidized *ent*-trachylobane **3** and the C-3 oxidized **1**. Additionally, we have shown that selective oxidation at C-15 and at C-20 is feasible. Interestingly, the oxidation at C-15 promoted an unprecedented ring-opening event to enable an entry point to *ent*-atisane natural products. With the advent of modern C–H oxidation methods, innovative bioinspired retrosynthetic bond disconnections that were previously impossible have found their way into natural product synthesis. This has inspired us to further explore the potential of C–H oxidation for the synthesis of structurally related natural product families. These studies are currently in progress in our laboratories and will be reported in due course.

## Supplementary Material

Supporting Information

## Figures and Tables

**Figure 1:**
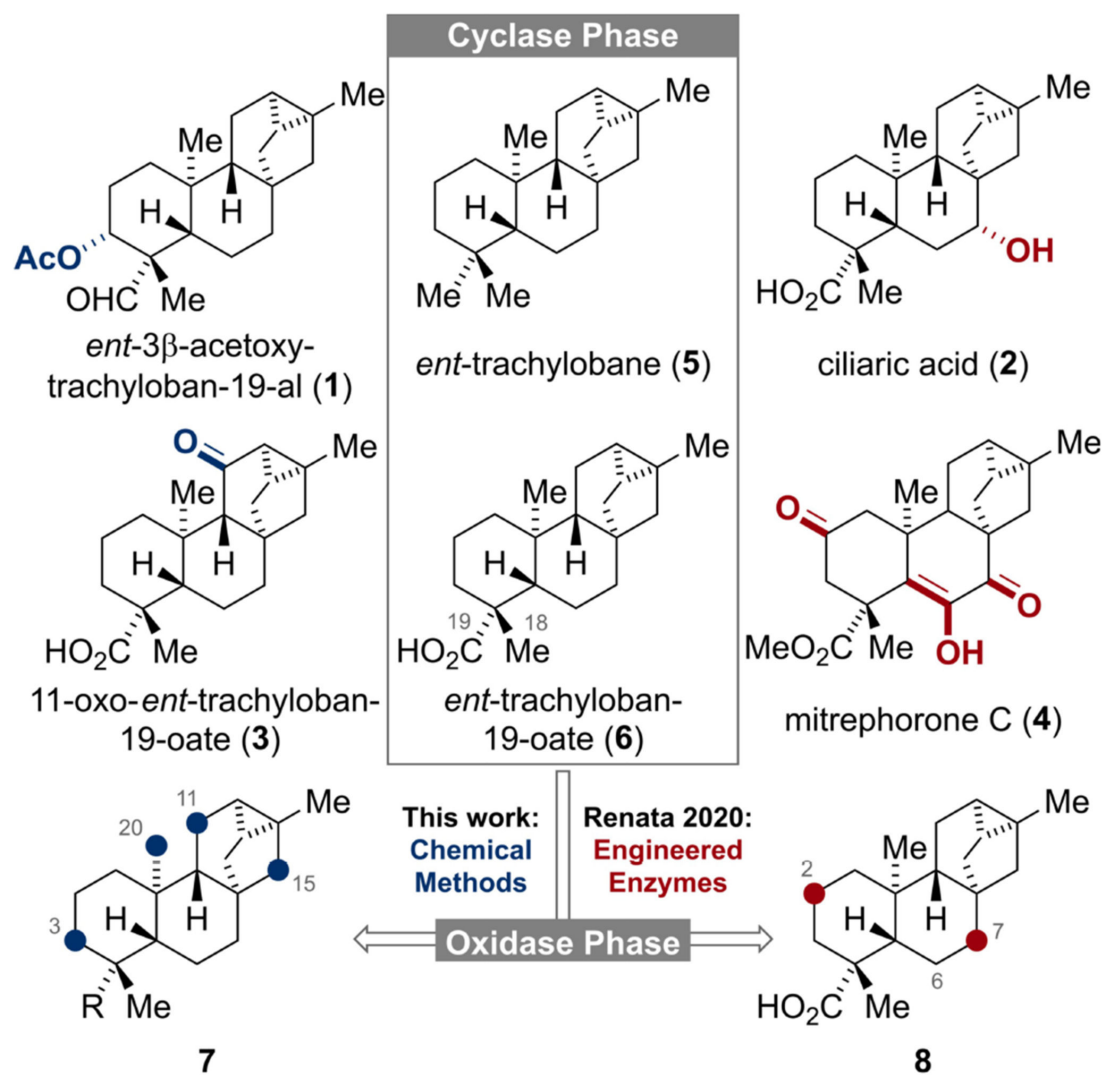
Structures of selected *ent*-trachylobanes and C-H oxidations of the carbon framework.

**Scheme 1:**
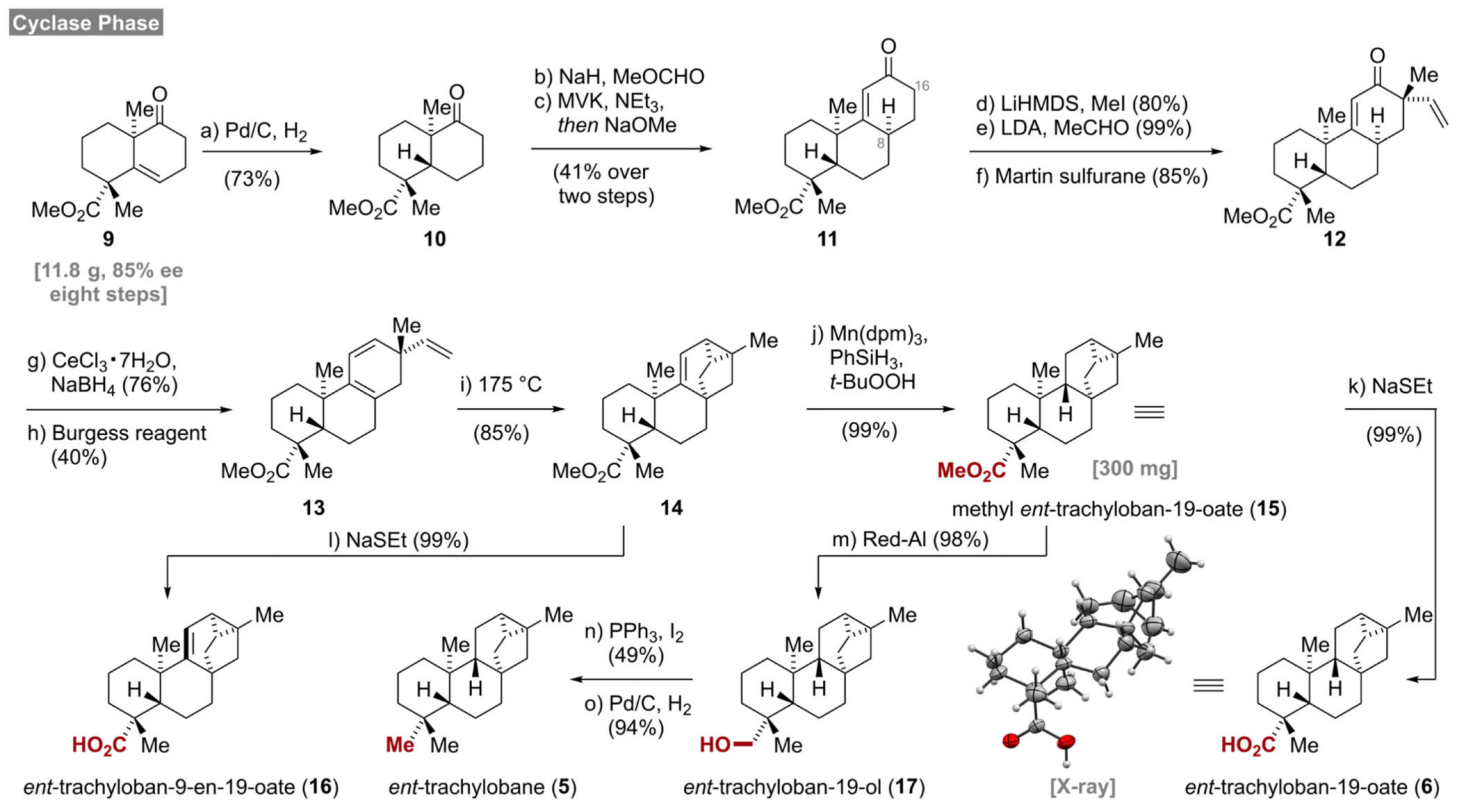
Total syntheses of *ent*-trachylobanes, reagents and conditions a) Pd/C, H_2_, AcOH, EtOAc, 23 °C, 8 d, 73 %; b) NaH, *then* MeOCHO, THF–PhMe, 0 °C to 23 °C, 4 h; c) MVK, NEt_3_, CH_2_Cl_2_, 23 °C, 48 h, *then* NaOMe, MeOH, 23 °C, 24 h, 41% over two steps; d) LiHMDS, *then* MeI, THF, -50 °C to 23 °C, 25 h, 80 %; e) LDA, TMEDA, *then* MeCHO, THF, –20 °C *then –78* °C, 3 h, 99 %; f) Martin sulfurane, PhH–CH_2_Cl_2_, 23 °C, 3 h, 85 %; g) NaBH_4_, CeCl3·7H_2_O, MeOH, 0 °C, 2 h, 76 %; h) Burgess reagent, DME, 75 °C, 2 h, 40 %; i) PhMe, 175 °C, 9 h, 85 %; j) Mn(dpm)_3_, PhSiH_3_, *t*-BuOOH, */*-PrOH, 23 °C, 4 h, 99 %; k) NaH, EtSH, DMF, 120 °C, 3 h, 99 %; l) NaH, EtSH, DMF, 120 °C, 4 h, 99 %; m) Red-Al®, PhMe, –20 °C to 23 °C, 22 h, 98 %; n) PPh_3_, I_2_, imidazole, PhMe, 60 °C, 3 h, 49 %; o) Pd/C, H_2_, NaOAc, MeOH, 23 °C, 16 h, 94 %.

**Scheme 2:**
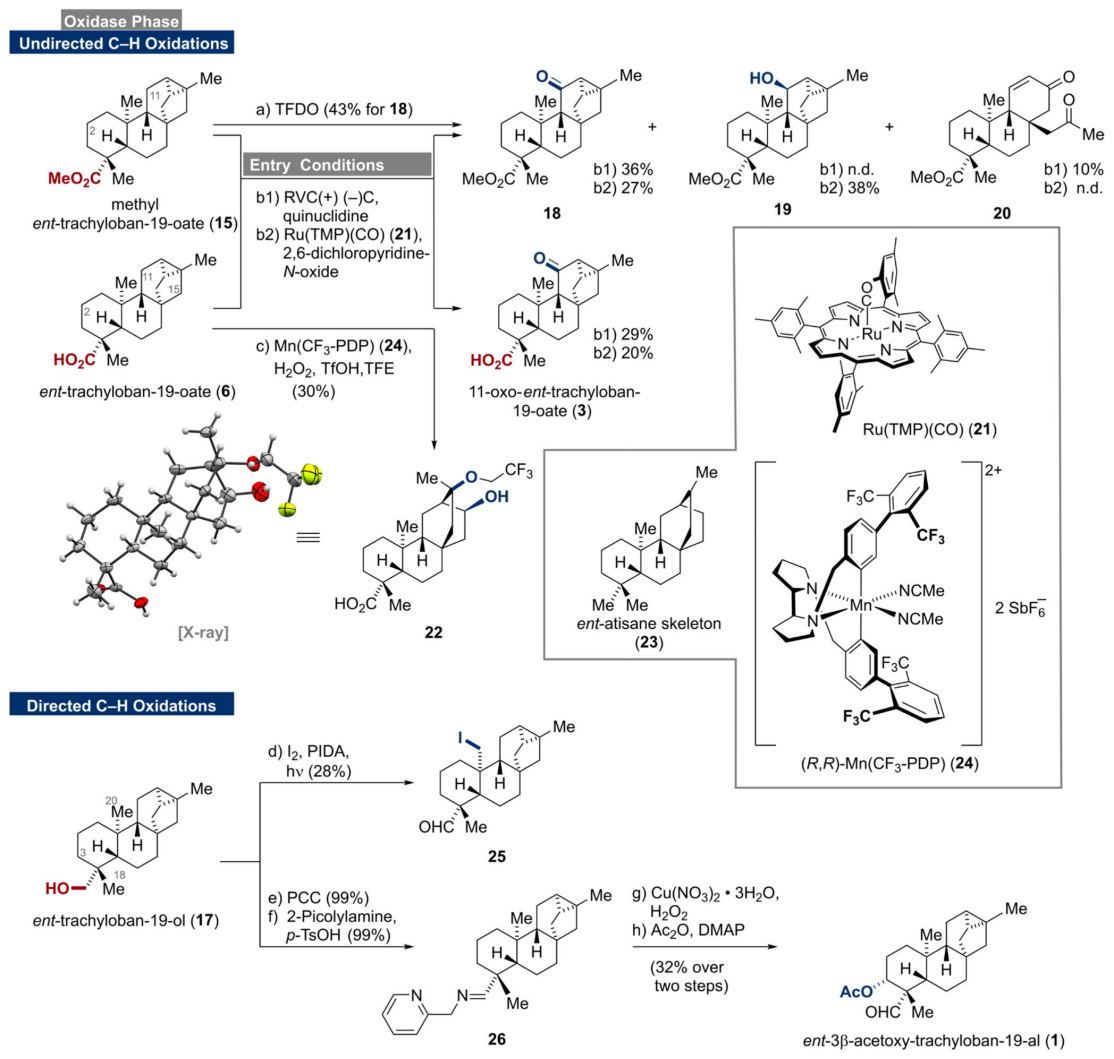
C–H oxidations of *ent*-trachylobanes **15**, **6**, and **17**, reagents and conditions a) TFDO, CH_2_Cl_2_, 0 °C, 3 h, 43 % for **18**; b1) (+)RVC foam/(-)Ni, Me_4_NBF_4_, quinuclidine, air, HFIP, MeCN, constant current, 23 °C, 36 % for **18**, 10 % for **20**, 29 % for **3**; b2) Ru(TMP)(CO) (**21**), 2,6-dichloropyridine-*N*-oxide, CH_2_Cl_2_, 65 °C, 27 % for **18**, 28 % for **19**, 20 % for **3**; c) (*R*,*R*)-Mn(CF3-PDP) (**24**), aq. H_2_O_2_, TfOH, TFE, 0 °C, 75 min, 30 %; d) I_2_, PIDA, *hν* (mercury vapor lamp), CyH, 23 °C, 2 h, 28 %; e) PCC, CH_2_Cl_2_, 0 °C to 23 °C, 3 h, 99 %; f) 2-picolylamine, *p*-TsOH ·H_2_O, PhMe, 80 °C, 16.5 h, 99 %; g) Cu(NO3)2·3 H_2_O, H_2_O_2_, THF, 50 °C, 4.5 h; h) Ac_2_O, DMAP, pyridine, 23 °C, 8 h (32 % over two steps).
